# Supplementary material for: Glucagon-Like Peptide-1 Receptor Agonists in Patients with Type 2 Diabetes: Prescription According to Reimbursement Constraints and Guideline Recommendations in Catalonia
Source: J Clin Med. 2019 Sep 5;8(9):1389. doi: 10.3390/jcm8091389 (PMC6780172; doi:10.3390/jcm8091389)
Supplement: Supplementary file 1 [file jcm-08-01389-s001.pdf]

Table S1. Mean change (SD) in clinical variables after 6 and 12 months according to subcategories of baseline HbA1c and BMI values

Clinical variable	Endpoint	Baseline HbA1c (%)				Baseline BMI (kg/m²)			
		<7% (n=271)	7-10 (n=1471)	>10 (n=464)	P-value	**<30 (n=201)**	**30-35 (n=734)**	**>35 (n=1369)**	P-value
HbA1c, %	*6 months*	0.02 (1.08)	-0.87 (1.41)	-2.20 (2.04)	**<0.001**	-0.54 (1.58)	-0.85 (1.59)	-0.89 (1.72)	0.097
	12 months	-0.02 (0.98)	-0.94 (1.32)	-2.22 (1.96)	**<0.001**	-0.73 (1.30)	-0.82 (1.56)	-0.92 (1.61)	0.358
BMI, kg/m²	*6 months*	-1.09 (2.61)	-1.02 (2.22)	-0.73 (2.05)	0.067	0.13 (2.13)	-0.61 (1.96)	-1.37 (2.50)	**<0.001**
	12 months	-1.12 (2.60)	-1.04 (2.00)	-1.04 (2.05)	0.838	0.22 (2.28)	-0.69 (1.68)	-1.46 (2.37)	**<0.001**
Weight, kg	*6 months*	-2.97 (7.28)	-2.69 (5.96)	-2.03 (5.53)	0.100	0.27 (5.85)	-1.71 (5.48)	-3.65 (6.74)	**<0.001**
	12 months	-3.05 (7.27)	-2.69 (5.29)	-2.70 (5.32)	0.618	0.53 (6.22)	-1.88 (4.65)	-3.84 (6.34)	**<0.001**
Total-Cholesterol, mg/dL	*6 months*	-5.50 (35.9)	-6.99 (34.7)	-9.88 (39.2)	0.269	-6.99 (35.5)	-5.40 (37.3)	-8.52 (36.3)	0.364
	12 months	-5.36 (35.7)	-7.13 (34.4)	-14.60 (36.7)	**0.016**	-6.60 (34.2)	-5.55 (35.5)	-10.24 (36.1)	0.177
HDL-Cholesterol, mg/dL	*6 months*	0.01 (6.98)	-0.03 (7.03)	0.36 (7.74)	0.737	0.26 (8.64)	0.32 (7.20)	0.08 (6.99)	0.854
	12 months	-0.03 (7.18)	-0.22 (6.93)	0.13 (7.36)	0.840	0.20 (6.43)	0.26 (7.32)	-0.18 (7.10)	0.662
LDL-Cholesterol, mg/dL	*6 months*	-4.93 (31.4)	-4.81 (29.9)	-6.89 (34.3)	0.624	-5.66 (32.7)	-3.52 (32.9)	-6.37 (31.3)	0.331
	12 months	-5.16 (31.2)	-4.96 (29.4)	-10.41 (32.0)	0.110	-4.43 (30.2)	-4.02 (31.7)	-7.57 (31.1)	0.261
Triglycerides, mg/dL	*6 months*	1.35 (96.8)	-12.06 (143)	-55.37 (273)	**<0.001**	-37.92 (213)	-13.15 (118)	-21.08 (183)	0.336

Clinical variable	Endpoint	Baseline HbA1c (%) <7% (n=271)	7-10 (n=1471)	>10 (n=464)	P-value	Baseline BMI (kg/m^2) <30 (n=201)	30-35 (n=734)	>35 (n=1369)	P-value
	12 months	-2.00 (94.3)	-12.60 (114)	-76.05 (280)	**<0.001**	-33.65 (199)	-18.10 (126)	-27.78 (167)	0.567
SBP, mmHg	*6 months*	-0.92 (17.4)	-0.85 (16.7)	-1.32 (19.1)	0.906	-0.25 (15.7)	-0.38 (16.5)	-1.21 (18.1)	0.552
	12 months	-1.42 (15.7)	-1.93 (15.6)	-3.45 (17.0)	0.262	-1.52 (13.2)	-1.63 (15.7)	-2.22 (16.0)	0.757
DBP, mmHg	*6 months*	0.24 (10.5)	-0.41 (9.88)	-0.81 (10.8)	0.269	-0.14 (9.78)	-0.36 (9.97)	-0.35 (10.8)	0.968
	12 months	-0.25 (9.37)	-1.03 (9.19)	-1.88 (9.50)	0.080	-0.85 (9.13)	-1.15 (8.99)	-1.04 (9.59)	0.944
Heart rate, bpm	*6 months*	-0.09 (12.5)	0.73 (12.5)	1.00 (12.8)	0.406	2.25 (11.5)	1.39 (12.8)	0.35 (12.7)	0.124
	12 months	-0.25 (9.37)	-1.03 (9.19)	-1.88 (9.50)	0.080	-0.85 (9.13)	-1.15 (8.99)	-1.04 (9.59)	0.944
eGFR, mL/min/1.73 m^2	*6 months*	-1.66 (11.6)	-1.25 (10.3)	-2.54 (11.9)	0.174	-2.49 (10.4)	-1.50 (10.7)	-1.58 (11.7)	0.668
	12 months	-1.31 (10.8)	-0.87 (9.80)	-1.90 (11.5)	0.392	-2.86 (10.8)	-0.68 (10.4)	-1.28 (11.1)	0.212

BMI, body mass index; DBP, diastolic blood pressure; eGFR, estimated glomerular filtration rate using the CDK-Epi equation; HbA1c, glycated haemoglobin A1c; SBP, systolic blood pressure; SD, standard deviation

Table S2. Goal achievement after 6 and 12 months of GLP-1RA initiation and according to prescription recommendations or local health policy for reimbursement.

			Prescribed according to SmPC?*			Prescribed according to reimbursement policy?**		
Goal	Endpoint	All population N=2854	Yes n=1721	No n=1133	P-value	Yes n=1493	No n=1361	P-value
HbA1c reducttion ≥1%	*6 months*	842 (43.5%)	741 (48.6%)	101 (24.7%)	**<0.001**	662 (50.0%)	180 (29.5%)	**<0.001**
	12 months	630 (43.1%)	548 (48.1%)	82 (25.5%)	**<0.001**	484 (48.8%)	146 (31.1%)	**<0.001**
Weight reduction ≥3%	*6 months*	900 (44.3%)	579 (44.3%)	321 (44.3%)	1.000	599 (44.8%)	301 (43.2%)	0.524
	12 months	657 (45.0%)	426 (44.7%)	231 (45.5%)	0.820	447 (45.5%)	210 (43.9%)	0.618
HbA1c reducttion ≥1% AND weight reduction ≥3%	*6 months*	336 (22.4%)	297 (25.0%)	39 (12.4%)	**<0.001**	302 (24.9%)	34 (11.7%)	**<0.001**
	12 months	201 (22.7%)	177 (25.4%)	24 (12.6%)	**<0.001**	181 (25.3%)	20 (11.6%)	**<0.001**
HbA1c reducttion ≥1% OR weight reduction ≥3%	*6 months*	1406 (57.1%)	1023 (62.2%)	383 (46.7%)	**<0.001**	959 (66.2%)	447 (44.0%)	**<0.001**
	12 months	1086 (53.3%)	797 (57.1%)	289 (45.2%)	**<0.001**	750 (59.5%)	336 (43.3%)	**<0.001**

*GLP-1RAs must me given in combination with other antidiabetics and in subjects poorly control (HbA1c ≥7%) and eGFR ≥45 mL/min/1.73 m^2
**GLP-1RAs must me given in combination with other antidiabetics and in subjects poorly control (HbA1c ≥7%) and BMI ≥30 kg/m^2
BMI, body mass index; HbA1c, glycated haemoglobin A1c; SmPC, summary of product characteristics

Table S3. Goal achievement after 6 and 12 months of GLP-1RA initiation and according to baseline HbA1c and BMI levels.

Goal	Endpoint	Baseline HbA1c (%)				Baseline BMI (kg/m²)			
		<7% **(n=271)**	7-10 **(n=1471)**	>10 **(n=464)**	**P-value**	<30 (n=201)	30-35 (n=734)	>35 (n=1369)	**P-value**
HbA1c reduction ≥1%	*6 months*	15 (6.5%)	572 (42.6%)	255 (71.2%)	**<0.001**	44 (35.5%)	230 (45.0%)	456 (45.0%)	0.121
	12 months	12 (6.7%)	429 (42.0%)	189 (72.1%)	**<0.001**	39 (39.8%)	161 (41.7%)	343 (45.0%)	0.426
Weight reduction ≥3%	*6 months*	84 (43.3%)	521 (44.7%)	127 (40.8%)	0.473	51 (30.9%)	258 (40.4%)	586 (48.0%)	**<0.001**
	12 months	60 (42.9%)	384 (44.4%)	95 (44.2%)	0.940	35 (29.9%)	188 (40.0%)	431 (49.6%)	**<0.001**
HbA1c reduction ≥1% AND weight reduction ≥3%	*6 months*	10 (5.81%)	243 (22.9%)	83 (30.6%)	**<0.001**	18 (16.8%)	86 (18.8%)	229 (24.6%)	**0.019**
	12 months	5 (5.00%)	149 (23.1%)	47 (33.3%)	**<0.001**	12 (18.5%)	43 (15.9%)	145 (26.5%)	**0.002**
HbA1c reduction ≥1% OR weight reduction ≥3%	*6 months*	89 (35.0%)	850 (58.6%)	299 (75.1%)	**<0.001**	77 (42.3%)	402 (58.0%)	813 (62.4%)	**<0.001**
	12 months	67 (30.6%)	664 (53.6%)	237 (70.5%)	**<0.001**	62 (41.3%)	306 (52.2%)	629 (58.0%)	**<0.001**

BMI, body mass index; HbA1c, glycated haemoglobin A1c

Table S4. Cumulative incidence of acute and chronic pancreatitis and thyroid cancer after 12 months of treatment initiation with a GLP-1RA

Adverse event (ICD-10 code)	All population (n = 2854)	Exenatide (n = 668)	Liraglutide (n = 1575)	Lixisenatide (n = 569)
Acute or chronic pancreatitis (K85 and K86), n (%)	11 (0.39%)	2 (0.30%)	8 (0.51%)	1 (0.18%)
Pancreatic cancer (C25), n (%)	5 (0.18%)	0 (0.0%)	4 (0.25%)	1 (0.18%)
Thyroid cancer (C73), n (%)	2 (0.07%)	1 (0.15%)	1 (0.06%)	0 (0.0%)

ICD-10, International Classification of Diseases

Table S5. Multivariate analyses on the probabilities to attain clinically beneficial goals after 6months of GLP-1RA treatment initiation

Baseline characteristic	Reduction in HbA1c ≥1%		Reduction in weight ≥3%		Reduction HbA1c ≥1% AND weight ≥3%		Reduction in HbA1c ≥1%, weight ≥3% OR both	
	OR (95 % CI)	p-value	OR (95 % CI)	p-value	OR (95 % CI)	p-value	OR (95 % CI)	p-value
**Gender**								
**Female**	Ref.		Ref.		Ref.		Ref.	
**Male**	1.16 (0.9-1.5)	0.301	0.82 (0.6-0.9)	0.118	1.12 (0.8-1.6)	0.491	0.84 (0.6-1.1)	0.170
**Age**	1.02 (1.0-1.04)	0.078	1.00 (0.99-1.02)	0.619	1.01 (0.99-1.0)	0.247	1.01 (1.0-1.0)	0.142
**T2D duration**								
**<5 ys**	Ref.		Ref.		Ref.		Ref.	
**5-10 ys**	0.89 (0.6-1.3)	0.577	1.22 (0.9-1.7)	0.259	1.48 (0.9-2.4)	0.089	0.86 (0.6-1.2)	0.391
**10-20 ys**	**0.64 (0.4-0.97)**	**0.036**	1.18 (0.8-1.7)	0.381	1.3 (0.8-2.0)	0.354	**0.67 (0.5-1.0)**	**0.031**
**>20 ys**	1.31 (0.6-2.6)	0.455	1.57 (0.8-2.9)	0.154	1.83 (0.8-4.0)	0.139	1.2 (0.6-2.4)	0.560
**HbA1c**	**2.06 (1.8-2.3)**	**<0.001**	0.92 (0.8-1.0)	0.067	**1.25 (1.1-1.4)**	**<0.001**	**1.41 (1.3-1.6)**	**<0.001**
**BMI**	1.02 (1.0-1.05)	0.145	**1.03 (1.01-1.05)**	**0.014**	**1.03 (1.00-1.06)**	**0.032**	**1.02 (1.0-1.1)**	**0.043**
**SBP**	0.996 (0.99-1.01)	0.375	1.01 (1.0-1.01)	0.210	1.00 (1.0-1.0)	0.632	1.0 (1.0-1.0)	0.877
**DBP**	1.01 (0.99-1.02)	0.347	1.00 (0.99-1.02)	0.628	1.01 (1.0-1.0)	0.612	1.0 (1.0-1.0)	0.377
**HDL-c**	1.01 (0.98-1.03)	0.588	1.00 (0.98-1.02)	0.981	1.00 (1.0-1.0)	0.753	1.0 (1.0-1.0)	0.341
**LDL-c**	1.00 (0.98-1.02)	0.951	1.00 (0.98-1.01)	0.719	1.00 (1.0-1.0)	0.685	1.0 (1.0-1.0)	0.748
**Total-c**	1.00 (0.98-1.02)	0.938	1.01 (0.99-1.02)	0.717	1.01 (0.99-1.0)	0.402	1.0 (1.0-1.0)	0.491
**Triglycerides**	1.00 (1.00-1.00)	0.589	1.00 (1.0-1.0)	0.717	1.00 (1.0-1.0)	0.567	1.0 (1.0-1.0)	0.905
**eGFR**	1.01 (1.0-1.02)	0.098	1.00 (0.99-1.01)	0.798	1.01 (1.0-1.0)	0.160	1.0 (1.0-1.0)	0.502

**Heart rate**	0.99 (0.98-1.00)	0.099	1.01 (1.00-1.02)	0.146	1.00 (1.0-1.0)	0.931	1.0 (1.0-1.0)	0.928
**Prescribed according to SmPC**	1.09 (0.6-1.95)	0.764	0.99 (0.6-1.6)	0.972	1.04 (0.5-2.1)	0.921	0.96 (0.6-1.6)	0.855
**Prescribed according HPR**	1.54 (0.9-2.6)	0.091	1.33 (0.9-2.1)	0.215	1.6 (0.8-3.0)	0.166	**1.6 (1.0-2.4)**	**0.031**

BMI, body mass index; DBP, diastolic blood pressure; eGFR, estimated glomerular filtration rate using the CDK-Epi equation; HbA1c, glycated haemoglobin A1c; HPR, health policy requirements for reimbursement; HR, heart rate; SBP, systolic blood pressure; SmPC, summary of product characteristics; TG, triglycerides

Table S6. Summary of the multivariate analyses on the probabilities to attain clinically beneficial goals after 6-12 months of GLP-1RA treatment initiation

Baseline characteristic	Reduction in HbA1c ≥1%		Reduction in weight ≥3%		Reduction HbA1c ≥1% AND weight ≥3%		Reduction in HbA1c ≥1%, weight ≥3% OR both	
	OR (95 % CI)	p-value	OR (95 % CI)	p-value	OR (95 % CI)	p-value	OR (95 % CI)	p-value
**Gender**								
Female	Ref.		Ref.		Ref.		Ref.	
Male	1.36 (1.0-1.9)	0.072	**0.73 (0.5-1.0)**	**0.036**	0.82 (0.5-1.2)	0.352	1.00 (0.8-1.3)	0.947
**Age**	1.02 (1.0-1.0)	0.117	1.01 (1.0-1.0)	0.189	1.02 (1.0-1.1)	0.165	1.01 (1.0-1-0)	0.205
**T2D duration**								
<5 ys	Ref.		Ref.		Ref.		Ref.	
5-10 ys	0.94 (0.6-1.5)	0.798	1.04 (0.7-1.6)	0.869	1.52 (0.8-2.9)	0.187	0.92 (0.6-1.3)	0.651
10-20 ys	0.69 (0.4-1.1)	0.143	1.12 (0.7-1.7)	0.645	1.30 (0.7-2.7)	0.316	0.82 (0.6-1.2)	0.320
>20 ys	1.88 (0.8-4.2)	0.121	1.39 (0.7-2.8)	0.369	**2.81 (1.0-7.6)**	**0.042**	1.54 (0.8-3.1)	0.201
**HbA1c**	**2.14 (1.9-2.5)**	**<0.001**	0.98 (0.9-1.1)	0.657	**1.42 (1.2-1.7)**	**<0.001**	**1.35 (1.2-1.5)**	**<0.001**
**BMI**	**1.04 (1.0-1.1)**	**0.015**	**1.04 (1.02-1.07)**	**0.001**	**1.06 (1.01-1.1)**	**0.003**	**1.03 (1.01-1.06)**	**0.005**
**SBP**	0.99 (0.98-1.0)	0.266	1.00 (1.0-1.0)	0.658	1.00 (1.0-1.0)	0.545	1.00 (1.0-1.0)	0.902
**DBP**	1.02 (1.0-1.0)	0.124	1.00 (1.0-1.0)	0.252	**1.03 (1.01-1.1)**	**0.018**	1.00 (1.0-1.0)	0.694
**HDL-c**	1.0 (1.0-1.0)	0.868	1.00 (1.0-1.0)	0.732	1.00 (1.0-1.0)	0.712	1.01 (1.0-1.0)	0.508
**LDL-c**	1.0 (1.0-1.0)	0.635	1.00 (1.0-1.0)	0.940	1.00 (1.0-1.0)	0.867	1.01 (1.0-1.0)	0.465
**Total-c**	1.0 (1.0-1.0)	0.680	1.00 (1.0-1.0)	0.985	1.00 (1.0-1.0)	0.669	1.00 (1.0-1.0)	0.448
**Triglycerides**	1.0 (1.0-1.0)	0.763	1.00 (1.0-1.0)	0.746	1.00 (1.0-1.0)	0.962	1.00 (1.0-1.0)	0.606
**eGFR**	1.0 (1.0-1.0)	0.080	1.00 (1.0-1.0)	0.332	**1.01 (1.0-1.03)**	**0.042**	1.00 (1.0-1.0)	0.363

**Heart rate**	1.0 (1.0-1.0)	0.532	1.00 (1.0-1.0)	0.532	1.00 (1.0-1.0)	0.410	1.00 (1.0-1.0)	0.670
**Prescribed according to SmPC**	1.23 (0.6-2.4)	0.532	1.18 (0.7-2.1)	0.577	1.53 (0.6-4.0)	0.372	1.10 (0.7-1.8)	0.723
**Prescribed according to HPR**	1.18 (0.7-2.1)	0.571	1.23 (0.7-2.1)	0.437	1.06 (0.5-2.4)	0.884	1.36 (0.9-2.1)	0.185

BMI, body mass index; DBP, diastolic blood pressure; eGFR, estimated glomerular filtration rate using the CDK-Epi equation; HbA1c, glycated haemoglobin A1c; HPR, health policy requirements for reimbursement; HR, heart rate; SBP, systolic blood pressure; SmPC, summary of product characteristics; TG, triglycerides
